# Mosquitoes and the city: effects of urbanization on *Aedes albopictus* and *Culex pipiens* captures in southern Spain

**DOI:** 10.1186/s13071-025-07094-2

**Published:** 2025-11-11

**Authors:** Mario Garrido, Paula Parra, Jesús Veiga, Marta Garrigós, Guillermo Panisse, Josué Martínez-de la Puente

**Affiliations:** 1 Biodiversity and Conservation Area, Rey Juan Carlos University, Móstoles , Madrid Spain; 2 Instituto de Investigación en Cambio Global (IICG-URJC), Rey Juan Carlos University, Móstoles, Madrid Spain; 3 Estación Biológica de Doñana (EBD, CSIC), Seville, Spain; 4 CIBER de Epidemiología y Salud Pública (CIBERESP), Madrid, Spain; 5 CEPAVE—Centro de Estudios Parasitológicos y de Vectores CONICET-UNLP, 1900 La Plata, Argentina

**Keywords:** Asian tiger mosquito, Urbanization, Invasive species, Mosquito surveillance, Seasonal abundance, Vector-borne diseases, Filarial nematodes

## Abstract

**Background:**

Urbanization and land-use changes profoundly affect mosquito ecology, potentially altering species’ abundance, seasonal dynamics, and pathogen transmission risk. The invasive mosquito *Aedes albopictus* has rapidly expanded from Southeast Asia to temperate regions worldwide, including Europe, where it now coexists with native species such as *Culex pipiens*. Both are competent vectors of zoonotic pathogens and may respond differently to urban environmental gradients.

**Methods:**

We assessed the impact of urbanization on mosquito populations by comparing the abundance and seasonality of *Ae. albopictus* and *Cx. pipiens* in urban and periurban areas of Granada, southern Spain, over two consecutive years (2023–2024). A total of 19 mosquito trapping sessions were conducted using BG-Sentinel traps baited with CO_2_ and BG-Lure, covering the main seasonal activity period. Additionally, 260 mosquito pools were screened for *Dirofilaria* spp. DNA using PCR.

**Results:**

A total of 450 *Ae. albopictus* and 641 *Cx. pipiens* females were captured. *Ae. albopictus* showed a unimodal seasonal pattern, peaking from late July to late August, with a more pronounced increase in urban sites. *Cx. pipiens* was more abundant in periurban areas, especially during its late July peak. Generalized linear mixed models revealed significant interactions between habitat and year for *Cx. pipiens*: abundance declined in periurban habitats from 2023 to 2024 but remained stable in urban areas. Seasonal peaks were also more extended in periurban sites. All mosquito pools tested negative for *Dirofilaria* spp.

**Conclusions:**

Our findings highlight species-specific responses to urbanization, with *Ae. albopictus* favoring urban environments and *Cx. pipiens* thriving in periurban zones. These patterns underscore the need for habitat- and species-specific vector surveillance and control strategies. Urban control efforts should target early summer *Ae. albopictus* peaks, particularly around artificial breeding sites, while periurban interventions should focus on *Cx. pipiens* populations that persist later in the season. The absence of *Dirofilaria* DNA suggests low current transmission, but continued molecular surveillance is warranted, particularly in periurban areas where high densities of *Cx. pipiens* vectors and animal reservoirs may overlap under changing environmental conditions.

**Graphical Abstract:**

**Figure:**
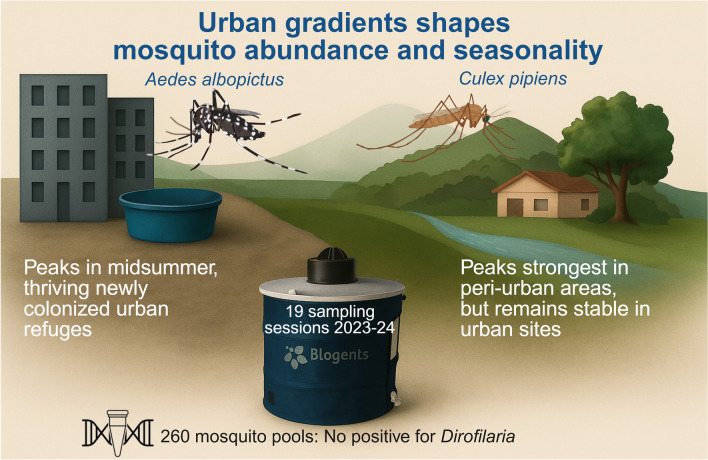

**Supplementary Information:**

The online version contains supplementary material available at 10.1186/s13071-025-07094-2.

## Background

Urban and periurban areas provide suitable environments for mosquito establishment and proliferation. Among urban species, the Asian tiger mosquito *Aedes albopictus*, listed among the world’s 100 worst invasive alien species, deserves special attention [1] due to its rapid expansion and significant impact on ecosystems and public health. Native from southern Asia, *Ae. albopictus* has increased its distribution range to other continents during the last decades from its native range to at least 28 other countries worldwide [2, 3]. In Europe, first detected in Albania in 1979, *Aedes albopictus* has spread to more than 15 countries [2, 3], including all of the Mediterranean Basin, from where it is now expanding northward, altering the epidemiological scenario of different pathogens in the areas [4].

Various pathogens of public health concern are now locally transmitted in parts of Europe previously free of competent vectors, creating novel epidemiological scenarios. The introduction and local proliferation of *Ae. albopictus* has enabled local transmission of imported arboviruses causing diseases, including dengue, chikungunya, and Zika, causing significant outbreaks in countries such as Italy, France, and Croatia in recent years [5–7]. In Spain, autochthonous dengue transmission has been confirmed, with *Ae. albopictus* from Catalonia testing positive in 2015 [8] and in recent locally detected human cases [9]. Besides viruses, *Ae. albopictus* is also a main vector of filarial nematodes (*Dirofilaria* spp.) from animal reservoirs to humans [4, 10], which caused outbreaks in Italy [10, 11].

The combination of high local densities of *Ae. albopictus*, the presence of infected hosts, and favorable ecological conditions have enabled these outbreaks. Contrary to the case of many other species, *Ae. albopictus* is positively affected by landscape anthropization considering urbanization, deforestation, and agricultural development [12]. Urban environments represent suitable areas for the maintenance of *Ae. albopictus* within the invaded regions, reaching high abundances in cities such as Barcelona [13]. Although natural breeding sites such as tree holes are uncommon in Europe [14], *Ae. albopictus* uses artificial containers and infrastructures, including sewers and ornamental fountains, for breeding in anthropized areas [15, 16]. In addition, humans, who are commonly found in urbanized areas, are common bloodmeal sources for this mosquito species [17]. Comparative studies reported differences in the abundances of *Ae. albopictus* between urban, periurban, and natural areas in China, with higher abundances in urban than in periurban and natural areas [16]. In northern Spain, ovitraps were used to compare the probability of *Ae. albopictus* occurrence across urban, suburban, and periurban environments [18]. The study found that *Ae. albopictus* was 4.39 times more likely to be detected in suburban areas than in periurban ones, with the highest probabilities recorded near parking lots. Similarly, research in Italy reported greater abundance of *Ae. albopictus* in highly urbanized areas compared with greener environments, although within the heavily urbanized Rome, the highest densities were found in small green areas [19]. These contrasting findings between studies suggest that fine-scale habitat characteristics can influence mosquito abundance and may help explain variability across studies. Despite the potential impact of habitat structure on *Ae. albopictus* population dynamics and the associated risk of pathogen transmission, studies addressing this topic in recently colonized areas remain limited.

Here, we sampled mosquitoes in three urban and three periurban areas of Granada, southern Spain, where *Ae. albopictus* is currently established [20], to assess the effect of habitat type on mosquito abundance. To account for temporal variation, mosquito sampling was conducted over two consecutive years. We also compared the abundance of *Ae. albopictus* with that of the native common house mosquito *Culex pipiens*, a widespread species in urbanized environments that often shares breeding sites—such as sewers—with *Ae. albopictus* [15]. *Culex pipiens* is a well-known vector of pathogens affecting humans, domestic animals, and wildlife in Spain, including West Nile virus, avian *Plasmodium*, and *Dirofilaria* parasites [21]. Thus, since both *Cx. pipiens* and *Ae. albopictus* are competent vectors of *Dirofilaria* spp. and considering the documented circulation of these parasites in Spain—including areas where *Ae. albopictus* populations are established, such as Barcelona [22]—we also conducted a molecular screening of the trapped mosquitoes to detect the presence of *Dirofilaria*. *Dirofilaria immitis* in domestic dogs from various Spanish localities ranges from 11.24% in Majorca to 2.63% in Madrid [23].

## Methods

### Mosquito sampling and study area

Mosquito sampling was conducted in the city of Granada and surrounding areas (southern Spain), during July–November 2023 (7 sessions) and March–November 2024 (12 sessions), with 1 session every 2–3 weeks. The metropolitan area of Granada is characterized by a semiarid Mediterranean climate with strong continental influences, featuring hot, dry summers and cold winters. Average annual precipitation ranges between 330 and 370 mm, concentrated mainly in autumn and spring, while summers are markedly arid. During the study period, temperature patterns varied significantly across sessions, whereas precipitation remained relatively stable, and neither showed significant differences between years (see *Supplementary Information* for further details).

**Figure 1:**
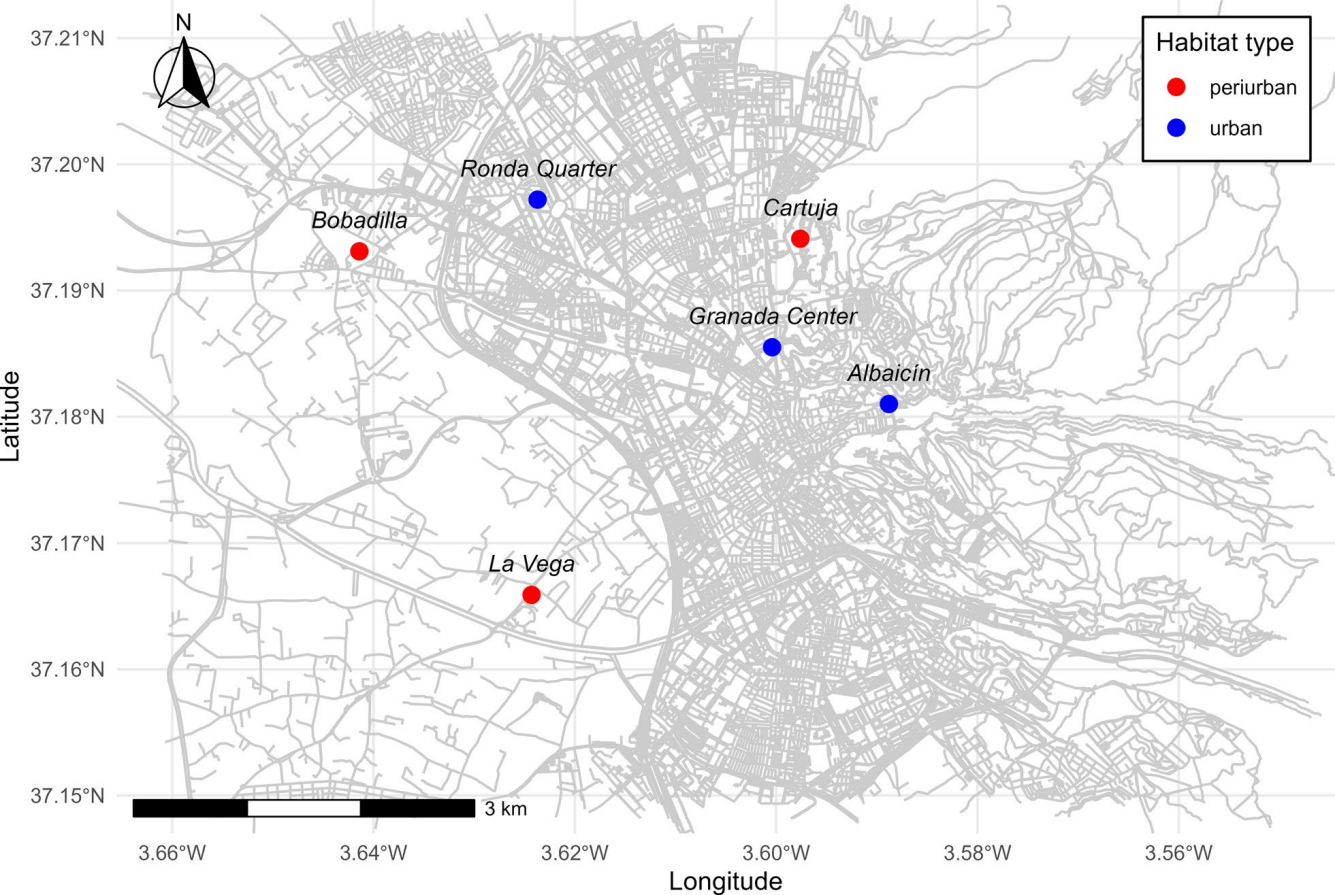
Mosquito sampling sites in the province of Granada, Spain. In blue, urban sampling sites: *Albaicín*, *Granada Center*, and *Ronda Quarter*. In red, periurban sampling sites: *La Vega*, *Bobadilla*, and *Cartuja*. Note that captures were conducted from July to November (7 sessions) in 2023 and from March to November in 2024 (12 sessions)

At each site, one BG-Sentinel-2 trap (Biogents, Germany) was operated for 24 h with dry ice (CO_2_) and BG-Lure as attractants; all traps were activated simultaneously across sites during each session. Six sampling sites were selected and classified into two habitat types—urban and periurban—on the basis of visual assessment of their landscape characteristics (Fig. 1). Urban sites were defined as areas within the central urban core, while periurban included natural/agricultural areas with limited urbanization and markedly lower population density, on the outskirts of the city [24]. The periurban sites included a natural area adjacent to a wastewater treatment facility surrounded by agricultural fields (*La Vega*; 37° 09′ 57.6" N, 3° 37′ 27.6" W); the grounds of a former sugar refinery now abandoned (*Bobadilla*; 37° 11′ 35.1" N, 3° 38′ 29.2" W); and a location with an intermediate level of urbanization on Cartuja campus of the University of Granada (*Cartuja*; 37° 11′ 38.7" N, 3° 35′ 51.2" W). Urban sites comprised a university guest residence with gardens located in Granada’s historic Albaicín district (*Albaicín*; 37° 10′ 51.5" N, 3° 35′ 19.8" W); the gardens of the Royal Hospital (*Granada Center*; 37° 11′7.9" N, 3° 36′ 1.6" W), a historic building in the city center; and the green areas within the School of Computer and Telecommunication Engineering, University of Granada, located in the Ronda district (*Ronda Quarter*; 37° 11′ 49.9" N, 3° 37′ 25.3" W). Further statistical analyses of 250 m buffers around sampling sites showed that urban areas had more urbanized land and higher population densities, whereas periurban areas had more natural land cover.

### Mosquito identification and molecular analyses

After collection, mosquitoes were transported to the laboratory on dry ice and subsequently sexed and identified to species level on the basis of their morphology [25]. For molecular screening of *Dirofilaria*, female *Ae. albopictus* and *Cx. pipiens* were grouped into pools of three to four individuals of the same species, site, and date of capture and stored at –80 °C until molecular analysis. Only females without visible signs of recent blood meals were included to prevent potential parasite amplification from ingested blood. Genomic DNA from each pool was extracted using the DNeasy® Blood & Tissue kit (Qiagen, Hilden, Germany). Samples were tested for the presence of *Dirofilaria* following the protocol used in Martínez-de la Puente et al. [21].

### Statistical analyses

All statistical analyses were conducted in R version 4.2.3 [26]. We restricted our analyses to female mosquitoes, given their higher abundances and greater epidemiological relevance. For each species (*Ae. albopictus* and *Cx. pipiens*), we fitted separate generalized linear mixed models (GLMMs) with a negative binomial distribution (family = nbinom2) and the total number of captured females as the response variable using the *glmmTMB* package [27]. The full model included year (categorical; 2023 versus 2024), habitat type (categorical; urban versus periurban), and capture date (continuous, expressed as Julian day; e.g., January 1 = 1, February 1 = 32), modeled as a second-degree polynomial, as fixed effects. We also considered all the two-way interactions among variables. To identify the seasonal dynamics more accurately, we modeled the effect of Julian day using a second-degree polynomial function. Sampling site was included as a random factor in the analyses to account for repeated sampling at the same sites.

Model selection followed a backward stepwise procedure. We sequentially removed nonsignificant interaction terms and main effects, starting from the full model and using Akaike’s Information Criterion (AIC) to select the most parsimonious model that adequately explained the data. Post hoc comparisons were conducted using estimated marginal means (EMMs) from the emmeans package [28]. When significant main effects or interactions were identified, pairwise contrasts were performed on the log-transformed response scale, with *P*-values adjusted for multiple comparisons using Tukey’s method.

## Results

Overall, 651 *Ae. albopictus* and 707 *Cx. pipiens* were captured. Females dominated mosquito captures, with 450 (177 in 2023, 273 in 2024) and 641 (434 in 2023, 207 in 2024) females captured corresponding to *Ae. albopictus* and *Cx. pipiens*, respectively (Table 1). Complementarily, we captured 76 individuals corresponding to other mosquito species, including *Culiseta longierolata* (*n* = 64), *Aedes caspius* (*n* = 2), *Culex* sp. (*n* = 2), and *Culiseta* sp. (*n* = 8).

**Table 1:** Number of *Aedes albopictus* and *Culex pipiens* females captured at each habitat type, categorized by year and sampling site

*Type of habitat*	Urban			Periurban			*Total*
*Sampling site*	Ronda Quarter	Granada Center	Albaicín	La Vega	Cartuja	Bobadilla	
*Aedes albopictus*							
* Total*	45	73	58	14	237	23	450
2023	20	30	36	5	83	3	177
2024	25	43	22	9	154	20	273
*Culex pipiens*							
*Total*	48	25	15	120	52	381	641
2023	10	9	6	69	32	308	434
2024	38	16	9	51	20	73	207

### Habitat effects on mosquito captures

Final GLMM for *Ae. albopictus* (conditional *R*^*2*^ = 0.67; marginal *R*^*2*^ = 0.54) included habitat type, sampling date, and their interaction. The habitat type × sampling date interaction was marginally significant (chi-squared test, *χ*^2^ = 5.51, *df* = 2, *P* = 0.06) but was retained in the model for its contribution to improved model fit on the basis of AIC values (450.8 with the interaction versus 452.4 without it). Sampling date main effect was highly significant (chi-squared test, *χ*^2^ = 56.52, *df* = 2, *P* < 0.001), while habitat type alone showed no significance (*χ*^2^ = 0.13, *df* = 1, *P* = 0.72). The year variable was excluded in previous steps due to its nonsignificance (*χ*^2^ = 2.14, *df* = 1, *P* = 0.15) and the higher AIC of the model including it (452.2 versus 450.8). Interestingly, the marginally significant habitat type × sampling date interaction term revealed that *Ae. albopictus* abundance followed a unimodal seasonal trend, peaking between late July and late August (Julian days 211–234), with the increase in mosquito abundance from early to peak season being more pronounced in urban areas than in periurban ones. After the peak, captures declined in both habitat types, reaching lower values at the end of sampling period in periurban areas than in urban ones.

**Figure 2:**
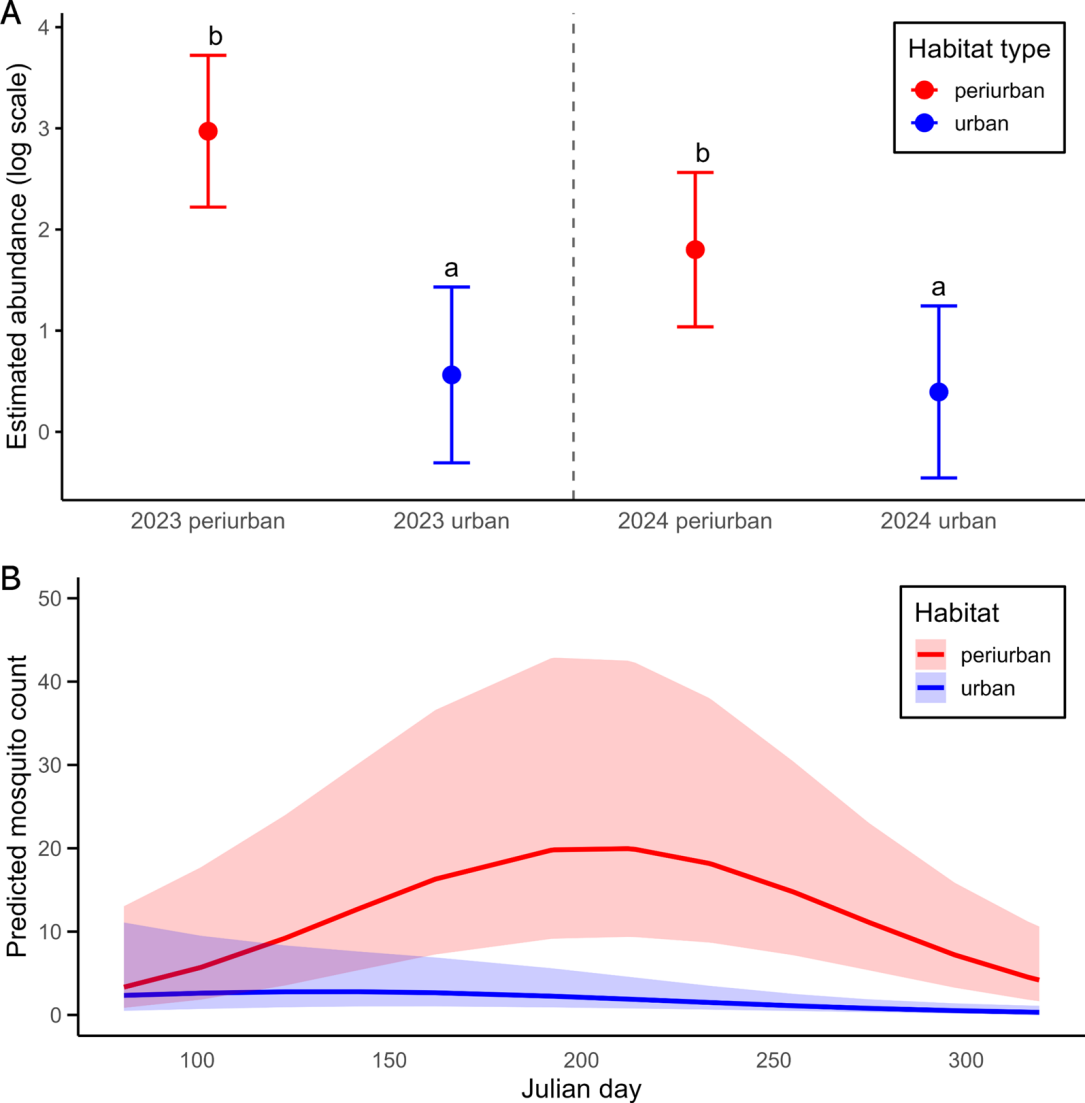
**A** Estimated mean abundance of *Culex pipiens* mosquitoes (± 95% CI) in urban and periurban habitats during 2023 and 2024, shown on the log scale of the model. Different letters indicate significant differences between pairs (Tukey-adjusted *P* < 0.05). **B** Seasonal variation in *Culex pipiens* mosquitoes across habitat types. Lines represent predicted mosquito abundances from a negative binomial GLMM (with 95% confidence ribbons) on the basis of the interaction between habitat type and Julian day

*Culex pipiens* final GLMM (conditional *R*^*2*^ = 0.87; marginal *R*^*2*^ = 0.73) included the significant effects of year (chi-squared test, *χ*^2^ = 12.11, *df* = 1, *P* < 0.001), habitat type (*χ*^2^ = 9.08, *df* = 1, *P* = 0.003), and sampling date (*χ*^2^ = 20.53, *df* = 2, *P* < 0.001), as well as the interactions between year × habitat type (*χ*^2^ = 3.94, *df* = 1, *P* = 0.047; Fig. 2A) and habitat type × sampling date (*χ*^2^ = 6.69, *df* = 2, *P* = 0.036; Fig. 2B). *Cx. pipiens* abundance was significantly higher in periurban than in urban areas during the two years of study (2023: estimate = 2.41 ± 0.59 SE, *z* = 4.11, *P* < 0.0001; 2024: estimate = 1.41 ± 0.58 SE, *z* = 2.41, *P* = 0.02). Yet, post hoc comparisons revealed that a significant interannual decline was observed in periurban habitats, with higher abundance in 2023 compared with 2024 (estimate = 1.17 ± 0.29 SE, *z* = 3.98, *P* = 0.0001), a pattern not found in urban areas (estimate = 0.17 ± 0.41 SE, *z* = 0.41, *P* = 0.68). Lastly, post hoc comparisons of the significant habitat type × sampling date interaction confirmed that mosquito abundance was significantly higher in periurban than in urban areas (estimate = 1.91 ± 0.53 SE, *z* = 3.62, *P* = 0.0003), especially around the seasonal peak (Fig. 2B; Julian day ~212, late July).

### *Dirofilaria* DNA detection

None of the 260 mosquito pools tested were positive for *Dirofilaria* DNA, while positive control samples were always amplified. Screened samples included 118 *Ae. albopictus* pools corresponding to 45 mosquito pools from urban areas (18 from Granada Center, 16 from Albaicín, and 11 from Ronda Quarter) and 73 mosquito pools from periurban sites (68 from Cartuja, 3 from Bobadilla, and 2 from La Vega). The remaining 142 mosquito pools tested corresponded to *Cx. pipiens*: 15 pools from urban areas (5 from Granada Center and 10 from Ronda Quarter) and 127 pools from periurban sites (11 from Cartuja, 88 from Bobadilla, and 28 from La Vega).

## Discussion

Our study examines how the degree of urbanization shapes the seasonal dynamics and habitat preferences of two key mosquito species in southern Spain: the invasive *Ae. albopictus* and the native *Cx. pipiens*. Although both species display broadly unimodal seasonal abundance patterns with overlapping peak periods, our results reveal contrasting habitat associations and temporal trends. These differences suggest distinct ecological responses of mosquitoes to urban versus periurban environments, which could affect the transmission of pathogens. While *Ae. albopictus* tended to reach different dynamics in both habitat types, *Cx. pipiens* abundance varied both temporally and spatially, following a broad unimodal seasonal pattern in both habitat types. However, urban sites exhibited earlier and sharper peaks in abundance, whereas periurban areas showed a more gradual increase and a slower decline, resulting in higher mosquito activity later in the season.

Different studies have identified contrasting effects of habitat anthropization on mosquito populations depending on the species. A recent meta-analysis revealed that, although higher levels of habitat degradation generally reduce mosquito captures, invasive *Aedes* species, including *Ae. albopictus*, may show the opposite trend [12]. In southern Spain, Ferraguti et al. [29] reported lower overall mosquito abundance in urban areas compared with rural and natural environments, a pattern that also applied to specific species. Thus, although *Cx. pipiens* was the most abundant species in urban settings among the 13 species surveyed, its abundance was significantly greater in natural habitats. In our study, *Cx. pipiens* was consistently more abundant in periurban than urban areas, with captures reaching higher values in 2023.

*Culex pipiens* had a seasonal peak in periurban sites that was more pronounced and extended over a longer period compared with urban areas. This is likely due to greater heterogeneity in water availability and vegetation cover of periurban sites, which could sustain higher mosquito densities for an extended part of the season [29, 30]. Urban populations, while still present, exhibited an earlier and less acute peak. These findings are consistent with previous research indicating that *Cx. pipiens* tends to exploit more rural or semi-natural breeding sites such as irrigation channels and ditches [30] and is more sensitive to interannual variation in climatic conditions or land-use changes, as suggested by the sharp decline in periurban captures in 2024. In this study, although temperature varied along the sampling period, temperature patterns were consistent between years (*Supplementary Information*), so this decline is more likely linked to rainfall variation and microhabitat conditions (e.g., irrigation events, temporary channels), including differences in water persistence, availability of refuges, and breeding sites. In contrast, mosquito abundances remained comparatively stable in urban areas, likely due to their more homogeneous landscapes and reduced sensitivity to environmental fluctuations.

Furthermore, we found that the abundance of *Ae. albopictus* peaked during the warmest period of the year (late July to late August), with a steeper increase in urban areas compared with periurban ones in southern Spain. Although the interaction between habitat and sampling date was only marginally significant, it did improve model fit and revealed an ecologically meaningful pattern: the urban population experienced a sharper rise and a more sustained abundance through the peak season, whereas periurban sites showed a smoother and transient increase. This finding is consistent with the established ecological knowledge of *Ae. albopictus* as a container-breeding species in invaded areas, which is closely associated with human-modified habitats. The proliferation of *Ae. albopictus* is likely to be facilitated by the presence of artificial water-holding human structures and higher densities and accessibility to human hosts [4, 30]. The apparent lack of a strong habitat effect in the main model term may reflect the high adaptability of *Ae. albopictus* to both habitat types in Mediterranean regions, but the interaction term supports the idea that urban microhabitats enhance early season population buildup. Interestingly, the highest captures of *Ae. albopictus* were obtained in the periurban site of Cartuja, comparable to that of urban sites (Table 1). This pattern may be explained by the fact that, despite its classification as a periurban area in the present study, Cartuja shares several features with the suburban environments as defined by Cevidanes et al. [18], such as the presence of built infrastructure and moderate human activity. Specifically, Cartuja hosts one of the University of Granada’s campuses, which, although not densely populated residentially, experiences high daily foot traffic and offers numerous artificial breeding sites, potentially supporting *Ae. albopictus* persistence.

Finally, despite the presence of competent vectors, the absence of *Dirofilaria* DNA in all mosquito pools tested suggests that transmission is occurring at very low levels or is absent in the study area, at least for the sampling sites considered. Although our sample size was considerable and representative of multiple sites and seasons, the negative results may be attributable to low local parasite prevalence or a high temporal and spatial variability in transmission [21, 31], as shown in various regions of Spain despite favorable climatic conditions and the presence of vectors [23]. Previous studies in the Iberian Peninsula have identified the presence of *Dirofilaria* and other parasite nematodes in *Cx. pipiens* mosquitoes [21, 32]. However, molecular screening for filarial worms in mosquitoes from other provinces in southern Spain revealed an extremely low parasite prevalence, with only two positive pools (one *Setaria equina* and one unidentified *Onchocerca* species) out of 1,282 tested pools comprising 22,791 female mosquitoes [31]. In addition, mosquitoes collected in areas of Barcelona, where *Dirofilaria* infections have been reported in humans, also tested negative [22]. This finding underscores the importance of longitudinal monitoring to detect temporal windows of transmission risk, particularly considering that both *Ae. albopictus* and *Cx. pipiens* are competent vectors of *D. immitis* under experimental and field conditions. [4, 11, 32].

## Conclusions

Our study has direct implications for mosquito surveillance and vector control programs. First, the clear differences in seasonal phenology and habitat preference between *Ae. albopictus* and *Cx. pipiens* call for species-specific strategies: while urban mosquito control efforts should focus on the summer increase in *Ae. albopictus* populations, particularly in artificial breeding sites, periurban interventions should target *Cx. pipiens* populations that persist later into the season. Second, the higher and more stable abundance of *Ae. albopictus* in urban areas highlights the importance of engaging municipalities and citizens in eliminating breeding habitats in densely populated zones. Finally, although no *Dirofilaria* DNA was detected, continued screening remains warranted to detect potential emergence events under changing climatic and ecological conditions, especially in periurban settings where vector densities and wildlife reservoirs might converge.

## Supplementary Information

Additional file 1.

## Data Availability

The datasets generated and/or analysed during the current study are available in the figshare repository at 10.6084/m9.figshare.29135129.
