# Supplementary material for: Mosquitoes and the city: effects of urbanization on Aedes albopictus and Culex pipiens captures in southern Spain
Source: Parasit Vectors. 2025 Nov 11;18:456. doi: 10.1186/s13071-025-07094-2 (PMC12606909; doi:10.1186/s13071-025-07094-2)
Supplement: Supplementary file 1 — Additional file 1. [file 13071_2025_7094_MOESM1_ESM.docx]

**SUPPLEMENTARY INFORMATION**

**Mosquitoes and the city: effects of urbanization on *Aedes albopictus* and *Culex pipiens* captures in southern Spain**

Mario Garrido^1,2*^, Paula Parra^3^, Jesús Veiga^4^, Marta Garrigós^3,4^, Guillermo Panisse^5^, Josué Martínez-de la Puente^3,4^

1. Biodiversity and Conservation Area, Rey Juan Carlos University, Móstoles, Madrid, Spain.
2. Instituto de Investigación en Cambio Global (IICG-URJC), Rey Juan Carlos University, Móstoles, Madrid, Spain.
3. Estación Biológica de Doñana (EBD, CSIC), Sevilla, Spain.
4. CIBER de Epidemiología y Salud Pública (CIBERESP), Madrid, Spain.
5. CEPAVE—Centro de Estudios Parasitológicos y de Vectores CONICET-UNLP, La Plata 1900, Argentina.

*Correspondence: Mario Garrido, [mario.garrido@urjc.es](mailto:mario.garrido@urjc.es); Josué Martínez-de la Puente, [jmp@ebd.csic.es](mailto:jmp@ebd.csic.es)

**Material and methods**

*Climatic data*

Climatic data for the seven days preceding each sampling session (Table S1) were extracted from the Granada Cartuja meteorological station (37°11'23.0"N, 3°35'44.0"W), the closest to all sampling sites, located approximately 3.7 km from *La Vega* (37°09'57.6"N, 3°37'27.6"W), the farthest sampling site. We fitted separate generalized linear models (GLMs) with 7-day averages of precipitation and mean, minimum, and maximum temperatures (sampling day plus the six preceding days) as response variables, and sampling session and year as predictors. These analyses showed that precipitation did not differ significantly across sessions (*F_11,6_* = 2.09, *p* = 0.19) or between years (*F_1,6_* = 0.06, *p* = 0.82). Similarly, mean, minimum, and maximum temperatures differed across sessions (mean: *F_11,6_* = 16.27, *p* = 0.001; minimum: *F_11,6_* = 18.22, *p* < 0.001; maximum: *F_11,6_* = 12.86, *p* < 0.01) but showed no significant interannual variation (mean: *F_1,6_* = 0.49, *p* = 0.51; minimum: *F_1,6_* = 0.67, *p* = 0.44; maximum: *F_1,6_* = 0.33, *p* = 0.59). These results indicate that while interannual variation was negligible, temperature fluctuated across sessions, whereas precipitation remained relatively stable. Due to the proximity of the sampling sites, no major variations in climatic conditions are expected between them; the maximum straight-line distance between any two sites was approximately 4.9 km between *Albaicín* (37°10'51.5"N, 3°35'19.8"W) and Bobadilla (37°11'35.1"N, 3°38'29.2"W).

**Table S1.** Sampling sessions with their corresponding dates and climatic conditions for 2023–2024. Mean, minimum, and maximum daily temperatures (°C) and precipitation (mm) are expressed as mean ± standard error, calculated over the sampling day and the six preceding days (n = 7 days). Note that in 2023, the first session (s6) was chosen to align session numbering with 2024 dates, ensuring that sessions with the same label correspond to comparable calendar periods in both years. Abbreviations: Temp., temperature; Precipit., precipitation.

| **Session** | **Sampling date** | **Mean temp.** (°C) | **Minimum temp.** (°C) | **Maximum temp.** (°C) | **Precipit.** (mm) |
| --- | --- | --- | --- | --- | --- |
| s6 | 12/07/2023 | 30.1±1.0 | 38.2±1.2 | 22.0±1.0 | 0.0±0.0 |
| s7 | 02/08/2023 | 28.9±0.4 | 37.3±0.6 | 20.5±0.4 | 0.0±0.0 |
| s8 | 21/08/2023 | 29.8±0.5 | 38.4±0.7 | 21.2±0.5 | 0.0±0.0 |
| s9 | 13/09/2023 | 24.9±0.5 | 32.3±0.6 | 17.6±0.4 | 0.0±0.0 |
| s10 | 02/10/2023 | 25.4±0.4 | 34.5±0.4 | 16.3±0.3 | 0.0±0.0 |
| s11 | 24/10/2023 | 16.1±0.9 | 20.8±1.2 | 11.4±0.7 | 5.6±4.2 |
| s12 | 15/11/2023 | 16.4±0.8 | 24.2±1.3 | 8.6±0.3 | 0.0±0.0 |
| s1 | 20/03/2024 | 17.7±1.0 | 25.3±1.1 | 10.1±1.1 | 0.1±0.1 |
| s2 | 10/04/2024 | 18.4±1.1 | 25.8±1.1 | 10.9±1.5 | 0.0±0.0 |
| s3 | 02/05/2024 | 11.8±0.5 | 16.9±0.9 | 6.7±0.5 | 5.3±2.2 |
| s4 | 21/05/2024 | 16.1±0.4 | 22.9±0.5 | 9.3±0.3 | 0.0±0.0 |
| s5 | 10/06/2024 | 23.1±1.1 | 30.7±1.6 | 15.5±0.6 | 0.6±0.3 |
| s6 | 09/07/2024 | 27.3±1.0 | 35.5±1.4 | 19.0±0.7 | 0.0±0.0 |
| s7 | 30/07/2024 | 31.8±0.4 | 40.8±0.3 | 22.8±0.5 | 0.5±0.5 |
| s8 | 21/08/2024 | 29.7±0.6 | 38.2±0.6 | 21.2±0.7 | 0.0±0.0 |
| s9 | 11/09/2024 | 23.8±0.4 | 31.8±0.7 | 15.9±0.4 | 2.3±2.1 |
| s10 | 30/09/2024 | 20.7±0.7 | 28.5±1.0 | 13.0±0.5 | 0.1±0.1 |
| s11 | 22/10/2024 | 18.0±1.2 | 24.1±1.8 | 11.8±1.2 | 1.5±1.4 |
| s12 | 11/11/2024 | 15.6±0.3 | 21.8±0.8 | 9.4±0.4 | 0.0±0.0 |
